# Red Blood Cell-Derived Iron Alters Macrophage Function in COPD

**DOI:** 10.3390/biomedicines9121939

**Published:** 2021-12-17

**Authors:** James M. Baker, Molly Hammond, Josiah Dungwa, Rajesh Shah, Angeles Montero-Fernandez, Andrew Higham, Simon Lea, Dave Singh

**Affiliations:** 1 Division of Immunology, Immunity to Infection and Respiratory Medicine, School of Biological Sciences, Faculty of Biology, Medicine and Health, Manchester Academic Health Science Centre, The University of Manchester and Manchester University NHS Foundation Trust, Manchester M13 9PL, UK; andrew.higham@manchester.ac.uk (A.H.); simon.lea@manchester.ac.uk (S.L.); dsingh@meu.org.uk (D.S.); 2 Medicines Evaluation Unit, Manchester University NHS Foundation Trust, Manchester M23 9QZ, UK; mollyhammond7@icloud.com (M.H.); jdungwa@meu.org.uk (J.D.); 3 Department of Thoracic Surgery, Manchester University Hospital NHS Foundation Trust, Manchester M13 9WL, UK; rajesh.shah@mft.nhs.uk; 4 Department of Histopathology, Manchester University Hospital NHS Foundation Trust, Manchester M13 9WL, UK; angeles.montero@mft.nhs.uk

**Keywords:** lung macrophage, iron, chronic obstructive pulmonary disease, phagocytosis

## Abstract

Lung macrophage iron levels are increased in COPD patients. Lung macrophage iron levels are thought to be increased by cigarette smoke, but the role of red blood cells (RBCs) as a source of iron has not been investigated. We investigate RBCs as a potential source of alveolar iron in COPD, and determine the effect of RBC-derived iron on macrophage function. We used lung tissue sections to assess RBC coverage of the alveolar space, iron and ferritin levels in 11 non-smokers (NS), 15 smokers (S) and 32 COPD patients. Lung macrophages were isolated from lung resections (*n* = 68) and treated with hemin or ferric ammonium citrate (50, 100 or 200 μM). Lung macrophage phenotype marker gene expression was measured by qPCR. The phagocytosis of Non-typeable *Haemophilus influenzae* (NTHi) was measured by flow cytometry. Cytokine production in response to NTHi in iron-treated macrophages was measured by ELISA. Lung macrophage iron levels were significantly correlated with RBC coverage of the alveolar space (*r* = 0.31, *p* = 0.02). Furthermore, RBC coverage and lung macrophage iron were significantly increased in COPD patients and correlated with airflow obstruction. Hemin treatment downregulated CD36, CD163, HLA-DR, CD38, TLR4, CD14 and MARCO gene expression. Hemin-treated macrophages also impaired production of pro-inflammatory cytokines in response to NTHi exposure, and decreased phagocytosis of NTHi (200 μM: 35% decrease; *p* = 0.03). RBCs are a plausible source of pulmonary iron overload in COPD. RBC-derived iron dysregulates macrophage phenotype and function.

## 1. Introduction

Chronic obstructive pulmonary disease (COPD) is characterised by airflow obstruction and persistent airway inflammation due to the inhalation of noxious gases such as cigarette smoke [1]. Cigarette smoking has been associated with increased pulmonary iron levels [2,3,4,5]. Increased levels of free iron in the lungs can generate harmful levels of reactive oxygen species (ROS) [6], contributing to inflammation and airway remodelling in COPD [7,8].

Lung macrophage numbers are increased in COPD patients, and show dysfunctional characteristics, including reduced phagocytosis, efferocytosis and cytokine production in response to bacteria [9,10,11]. Lung macrophages play an important role in the uptake of iron, including free iron and transferrin bound iron, in the lungs [5]. Intra- and extra-cellular iron can be stored as ferritin, which reduces free iron induced oxidative stress [12]. Both lung macrophage iron levels and extracellular ferritin levels are increased in COPD [5,13].

Multiple studies have focused on cigarette smoke as a source of increased pulmonary iron levels in COPD [4,5,14,15,16]. However, Corhay et al., stated that increased lung macrophage iron in COPD cannot solely be attributed to iron from cigarette smoke [2]. Red blood cells (RBCs) are rich in the iron-containing molecule heme [17]. Increased leakage of RBCs into the alveolar space in COPD, due to mechanisms such as vascular remodelling and pulmonary hypertension, may cause lung macrophage iron loading as RBCs undergo phagocytosis by macrophages [18]. Both free iron and heme can cause macrophage dysfunction, but the specific effects of these distinct iron sources differ [19,20,21]; for example, hemin inhibits phagocytosis [19,20,21], while equimolar concentrations of free iron do not [21]. The source of excess iron in the lungs is, therefore, an important potential determinant of macrophage dysfunction.

The aim of this study was to use COPD primary cells to study the importance of RBC-derived iron as a modulator of lung macrophage function. First, we investigated the relationship between RBC coverage and iron levels in COPD lung macrophages. Second, we investigated the effects of free iron versus hemin on COPD lung macrophage function. This study provides insights into a previously underappreciated source of lung iron in COPD and demonstrates the consequences on macrophage function.

## 2. Materials and Methods

### 2.1. Study Subjects

We recruited patients undergoing lung surgery for suspected cancer to collect lung tissue (*n* = 58). Lung tissue from a further 68 patients with suspected lung cancer was used to obtain lung macrophages. The COVID-19 pandemic stopped access to lung surgical samples. We, therefore, recruited 6 non-smokers and 10 COPD patients who volunteered to donate peripheral blood so that we could use monocyte-derived macrophages (MDMs) as an alternative. COPD was diagnosed based on a smoking history of ≥10 pack years and GOLD criteria [1]. Controls were smokers (S) without airflow limitation or non-smokers (NS). Ex-smokers were defined as individuals who had stopped smoking for ≥1 year. Research was approved by the NRES Committee North West-Greater Manchester South (reference 03/SM/396) and North West-Haydock (reference 20/NW/0302). This research was carried out in accordance with the World Medical Association Declaration of Helsinki, and all subjects provided written informed consent.

### 2.2. Lung Tissue Preparation

Lung tissue from patients was obtained from areas of the resected tissue most distal from the tumour, as previously described [22]. Tissue was formalin fixed and paraffin embedded (*n* = 58, Table S1). Tissue sections were analysed for airway red blood cell quantification, macrophage levels of ferritin and iron (described online). Tissue sections were stained with H&E. Ten random fields of view were used for analysis to calculate the percentage of the alveolar space covered with RBCs. ImagePro Plus 5.1 software (Media Cybernetics, Rockville, MD, USA) was used to measure the alveolar area (AA) and RBC area (RBC-A), as shown in a representative image (Figure S1). The alveolar space occupied by RBCs was then calculated using the following formula: alveolar space occupied by RBCs = (RBC-A/AA) × 100. Macrophage iron levels were quantified using a previously described semi-quantitative scale, Golde score [23]. The Golde score was calculated through assigning a staining intensity score (0–4) for each macrophage using the scale shown in Figure S2. Due to the ubiquitous expression of ferritin in lung macrophages, ferritin levels were assessed based on the intensity of staining to define lung macrophages as ferritin^high^ or ferritin^low^. Representative images of staining are shown online (Figure S3). CD68 staining was used to determine the quantity and distribution of lung macrophages.

### 2.3. Lung Macrophage Culture

Lung macrophages were isolated by perfusion of the airways of lung tissue as previously described [22], and outlined in the Supplementary Materials. Where indicated, lung macrophages were treated with 50, 100 or 200 μM of ferric ammonium citrate (FAC) or hemin or left untreated for 6 or 24 h. Cells were harvested for qPCR analysis of iron metabolism genes (6, 24 h) or CD163 release (24 h). The full methods are shown online.

### 2.4. Monocyte-Derived Macrophage Culture

MDMs were generated with culture for 7 days in GM-CSF (10 ng/mL) and treated with hemin or FAC (50, 100 or 200 μM) for 24 h.

Cells were then analysed for phagocytosis of heat-killed Non-typeable *Haemophilus influenzae* (NTHi), surface expression levels of TLR4 and CD14 protein by flow cytometry (gating strategy shown in Figures S4 and S5, respectively) or pro-inflammatory cytokine production (TNF-α, IL-6 and CXCL8) in response to NTHi exposure for a further 24 h and measured by ELISA (full methods described online).

### 2.5. Statistics

All statistical analyses were performed using GraphPad (GraphPad Prism version 9.0.1, GraphPad Software, La Jolla, CA, USA). Data testing for normality was determined by the D’Agostino and Pearson normality test. Parametric data were compared using a one-way ANOVA followed by Tukey’s post hoc analysis or an un/paired *t* test, where only two groups were compared. Non-parametric data were compared using a Kruskal–Wallis test followed by a Dunn’s post hoc analysis or Mann–Whitney test, where only two groups were compared.

## 3. Results

### 3.1. Study Subjects

In total, 142 patients were recruited to this study with clinical characteristics shown in Table 1. A further breakdown of clinical characteristics for each set of experiments is shown in Supplementary Tables S1–S8.

### 3.2. Lung Macrophage Iron and Ferritin Staining, and Alveolar RBC Coverage

**Figure 1:**
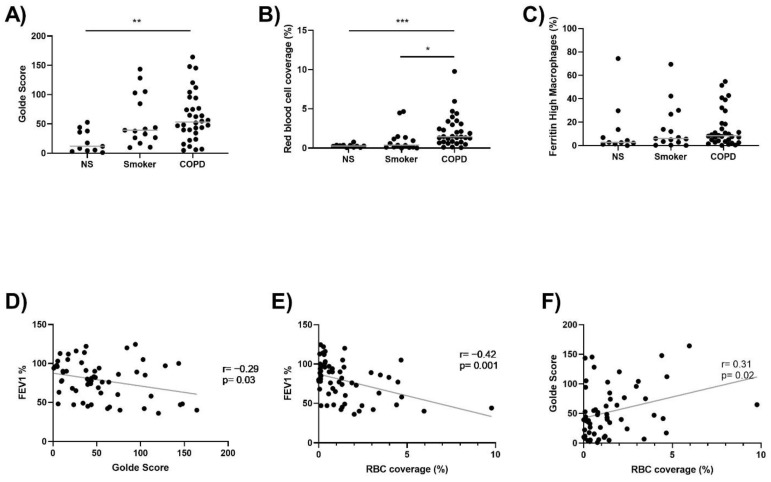
Lung macrophage iron levels and RBC coverage is increased in COPD. (**A**) Lung tissue sections were stained with Perls Prussian Blue. Lung macrophage iron levels were quantified in 11 NS, 15 S and 32 COPD patients. (**B**) H&E staining was carried out to assess RBC coverage in the alveolar space in 11 NS, 15 S and 32 COPD patients. (**C**) The percentage of ferritin^high^ lung macrophages in 11 NS, 15 S and 32 COPD patients. (**D**) Correlation between FEV1% and Golde score (*r* = −0.29, *p* = 0.03). (**E**) Correlation between FEV1% and RBC coverage of the alveolar space (*r* = −0.42, *p* = 0.001). (**F**) Correlation between Golde score and RBC coverage of the alveolar space (*r* = 0.31, *p* = 0.02). Kruskal–Wallis multiple comparisons test was used to test differences between groups. Correlation data are plotted as individuals with linear regression (**D**–**F**). * = *p* < 0.05, ** = *p* < 0.01, *** = *p* < 0.001. Data presented as individuals with median.

Lung macrophage iron (Golde score) and ferritin staining, and the presence of RBCs in the alveolar space, was determined in NS (*n* = 11), S (*n* = 15) and COPD patients (*n* = 32). Lung macrophage iron staining was numerically highest in COPD patients, with statistical significance compared to NS (*p* = 0.003) (Figure 1A). RBC coverage of the alveolar space was significantly increased in COPD patients compared to both S (*p* = 0.01) and NS (*p* = 0.0002) (Figure 1B). There were no differences between groups for the percentage of ferritin^high^ lung macrophages (Figure 1C). Lung macrophage iron staining and RBC coverage were both negatively correlated with FEV1% predicted (*p* = 0.03, *r* = −0.29, Figure 1D and *p* = 0.001, *r* = −0.42, Figure 1E, respectively). Lung macrophage iron staining was also significantly correlated with RBC coverage (*p* = 0.02, *r* = 0.31, Figure 1F).

### 3.3. Iron Metabolism Gene Expression in Lung Macrophages

**Figure 2:**
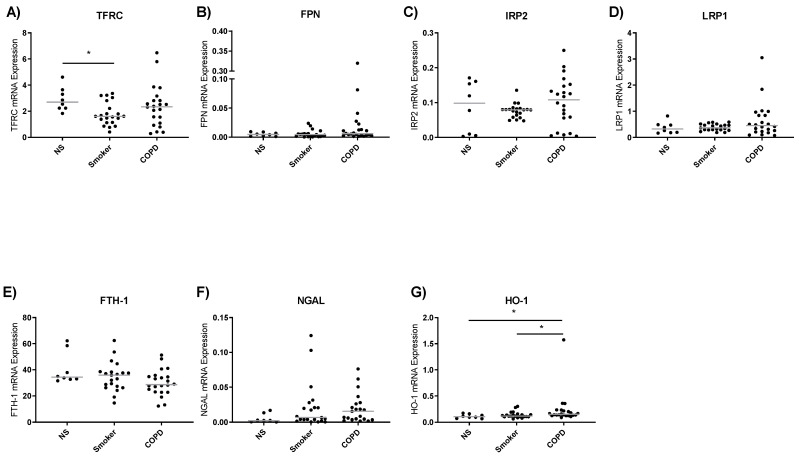
Baseline lung macrophage iron metabolism gene expression. Real-time qPCR was used to assess levels of TFRC, FPN, IRP2, LRP1, FTH-1, NGAL and HO-1 expression of lung macrophage from non-smoker (NS) (*n* = 8), smoker (S) (*n* = 24) and COPD (*n* = 27) (**A**–**G**). Expression levels are relative to GAPDH (2−^ΔCt^). Data presented as individuals with median. Kruskal–Wallis multiple comparisons test was used to test differences between groups. * = *p* < 0.05.

The expression of genes involved in cellular iron metabolism were assessed in lung macrophages from NS (*n* = 8), S (*n* = 24) and COPD patients (*n* = 27) (Figure 2). The expression of TFRC was significantly lower in S compared to NS (*p* = 0.04, Figure 2A), but there were no other differences between groups for this gene or FPN, IRP2, LRP1, FTH-1 and NGAL (Figure 2B–F). The expression of HO-1, which is involved in heme metabolism, was significantly increased in COPD lung macrophages compared to both NS and S controls (*p* = 0.02, *p* = 0.047, respectively, Figure 2G).

### 3.4. Effect of Iron Treatment on Lung Macrophage Gene Expression

The effects of treating lung macrophages with iron using either ferric ammonium citrate (FAC) or RBC-derived iron (hemin) (50, 100 or 200 μM) were evaluated, with gene expression measured at 6 and 24 h. Hemin and FAC treatments had no cytotoxic effects on lung macrophages (*p* > 0.05, Figure S6) and did not change the expression levels of pro-/anti-apoptosis genes in either COPD patients or S (Figure S7).

#### 3.4.1. Iron Metabolism Gene Expression

Hemin treatment significantly upregulated HO-1 expression at 6 and 24 h, with relative expression values of 7 and 10, respectively (Figure S8A). FPN was significantly downregulated by hemin treatment at 24 h (relative expression = 0.5, Figure S8C). Hemin treatment had no effect on FTH-1, IRP1 or IRP2 expression (Figure S8B,D,E). FAC treatment downregulated FTH-1 (at 24 h) and IRP1 (at 6 h) (Figure S8F,H), with no effect on FPN or IRP2 expression (Figure S8G,I).

#### 3.4.2. Inflammatory Gene Expression

**Figure 3:**
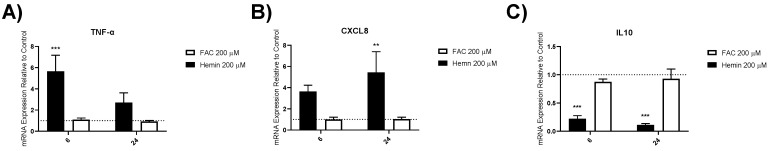
Inflammatory marker gene expression in iron-treated lung macrophages. TNF-α, CXCL8 and IL10 expression in lung macrophages treated with 200 μM of hemin or ferric ammonium citrate (FAC) (**A**–**C**). Real-time qPCR was used to assess levels of gene expression after 6 and 24 h. Gene expression is plotted as means relative to GAPDH and untreated control for each patient (2^−ΔΔCt^) ±SEM in smokers and COPD patients (*n* = 6). Dotted line at 1 indicates untreated control. One-way ANOVAs were carried out with a post hoc Dunnett’s multiple comparisons test against time-matched untreated controls. ** = *p* < 0.01, *** = *p* < 0.001.

Hemin treatment upregulated TNF-α and CXCL8 expression (Figure 3 shows results using 200 μM; lower concentrations shown in online Supplementary Materials, Figure S9), with TNF-α showing greater upregulation at 6 h (relative expression = 5.7, *p* = 0.0001) compared to 24 h, while CXCL8 showed greater upregulation at 24 h (relative expression = 5.4, *p* = 0.003). IL10 was significantly downregulated by hemin treatment (relative expression = 0.1, *p* < 0.0001). FAC treatment did not modulate TNF-α, CXCL8 or IL10 expression (Figure S10, *p* > 0.05).

#### 3.4.3. Macrophage Polarisation Marker Gene Expression

**Figure 4:**
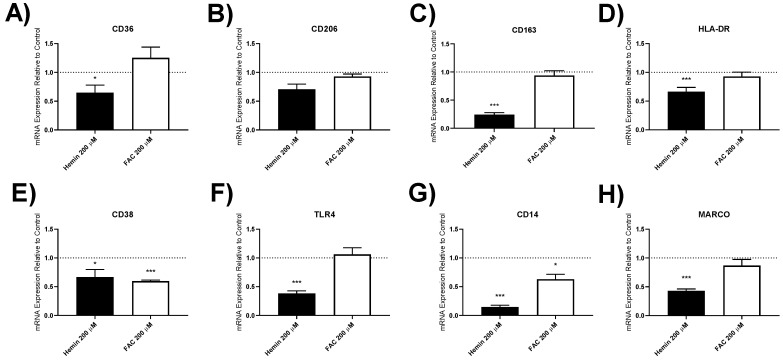
Macrophage polarisation markers in iron-treated lung macrophages. CD36, CD206, CD163, HLA-DR, CD38, TLR4, CD14, and MARCO expression in lung macrophages treated with 200 μM of hemin or ferric ammonium citrate (FAC) (**A**–**H**). Real-time qPCR was used to assess levels of gene expression after 24 h. Gene expression shown was plotted as means relative to GAPDH and an untreated control for each patient (2^−ΔΔCt^) ±SEM in smokers and COPD patients (*n* = 6). Dotted line at 1 indicates untreated control. One-way ANOVAs were carried out with a post hoc Dunnett’s multiple comparisons test against time-matched untreated controls. * = *p* < 0.05, *** = *p* < 0.001.

The expression of 8 genes involved in macrophage function (CD36, CD206, CD163, HLA-DR, CD38, TLR4, CD14 and MARCO) were assessed in response to hemin and FAC treatment (Figure 4 shows results using 200 μM at 24 h). Hemin significantly downregulated the expression of all of these genes except CD206 (Figure 4). There was also suppression of gene expression at 6 and 24 h with lower hemin concentrations (shown in the on-line Supplementary Materials, Figure S11).

FAC significantly decreased the expression of CD38 and CD14 at 24 h, with no effect on the other genes studied at 24 h (Figure 4 and online Supplementary Materials, Figure S12).

### 3.5. sCD163 Release from Iron-Treated Lung Macrophages

**Figure 5:**
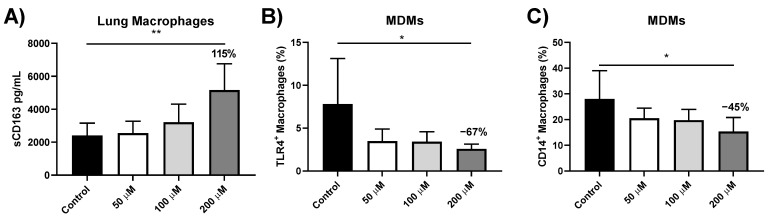
Protein levels in hemin-treated macrophages. (**A**) sCD163 levels in supernatants from lung macrophages treated with 50, 100 or 200 μM of hemin. ELISA was used to assess levels of sCD163 after 24 h in smokers and COPD patients (*n* = 8). Percentage of (**B**) TLR4 and (**C**) CD14-positive MDMs after 24 h treatment with 50, 100 or 200 μM of hemin in COPD patients (*n* = 5). One-way ANOVAs were carried out with a post hoc Dunnett’s multiple comparisons test against time-matched untreated controls. Data presented as ±SEM. Relative percentage changed indicated on graph. * = *p* < 0.05, ** = *p* < 0.01.

To determine whether decreased CD163 gene expression was associated with the increased cell surface cleavage of CD163, supernatant sCD163 protein levels following iron treatment were measured by ELISA. sCD163 levels were significantly increased by approximately 115% following 24 h treatment with hemin at 200 μM (*p* = 0.002, Figure 5A). FAC treatment did not significantly alter sCD163 levels (Figure S13A).

### 3.6. Effect of Iron Treatment on TLR4 and CD14 Protein Expression in MDMs

To determine if TLR4 and CD14 were modulated at the protein level following iron treatment, MDMs were treated with hemin or FAC for 24 h with TLR4 and CD14 levels analysed by flow cytometry (*n* = 5). Hemin treatment significantly decreased the percentage of MDMs expressing TLR4 with a 67% decrease at 200 μM (*p* = 0.02) (Figure 5B), and a decrease in median fluorescent intensity (MFI) levels was also observed (Figure S14A). Hemin also significantly decreased the percentage of MDMs expressing CD14 (45% decrease at 200 μM, *p* = 0.02) (Figure 5C) and MFI of CD14 (Figure S14C). FAC treatment showed no consistent changes in TLR4 and CD14 expression (described in online Supplementary Materials, Figures S13 and S14).

### 3.7. Effects of Iron Treatment on MDM Phagocytosis of NTHi

**Figure 6:**
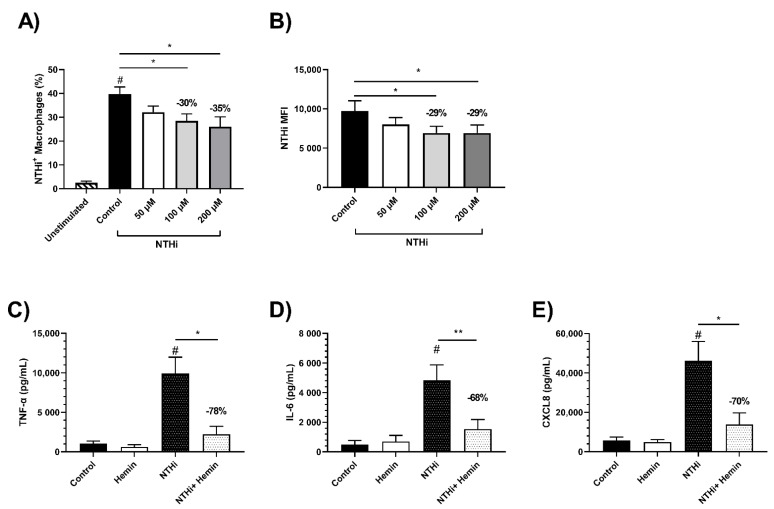
Phagocytosis and cytokine response following NTHi stimulation in hemin-treated MDMs. Monocyte-derived macrophages (MDMs) were pre-treated with 50, 100 or 200 μM of hemin for 24 h followed by exposure to labelled Non-typeable *Haemophilus influenzae* (NTHi) for 3 h. (**A**) Phagocytosis of FITC labelled NTHi in hemin pre-treated COPD MDMs was measured using flow cytometry. (**B**) Median fluorescent intensity (MFI) of COPD MDMs with labelled NTHi internalised. MDMs from COPD patients and non-smokers were pre-treated with 200 μM of hemin for 24 h and were subsequently stimulated for a further 24 h with live bacteria (*n* = 7). Cytokine production was measured for (**C**) TNFα, (**D**) IL6 and (**E**) CXCL8 in COPD patients and non-smokers. One-way ANOVAs were carried out with a post hoc Dunnett’s multiple comparisons test against untreated/stimulated controls (**A**,**B**). Relative percentage changed indicated on graph. * = *p* < 0.05, ** = *p* < 0.01. Unstimulated vs. stimulated control *t*-test # = *p* < 0.05.

We showed that MARCO gene expression is significantly downregulated following hemin treatment. Previously, MARCO downregulation has been shown to impair phagocytosis [24]. To determine whether hemin treatment would result in impaired phagocytosis of NTHi, MDMs from COPD patients (*n* = 7) and NS (*n* = 6) were treated with hemin or FAC (50, 100 or 200 μM) for 24 h followed by exposure to NTHi. Flow cytometry was used to assess NTHi phagocytosis. MDMs from both COPD patients and NS exposed to NTHi had significantly increased intracellular levels of NTHi compared to unexposed controls (# = *p* < 0.0001 for both, Figure 6A and Figure S14A).

Treatment of COPD MDMs with hemin significantly decreased the percentage of NTHi-positive MDMs; for example, 200 μM caused a 35% decrease (*p* = 0.03), with a similar change in MFI (29% decrease; *p* = 0.01; Figure 6A,B). Similar results were observed in MDMs from NS (described in the online Supplementary Materials, Figure S15).

Treatment of COPD or NS MDMs with FAC did not reduce macrophage phagocytosis (Figures S16 and S17).

### 3.8. Effect of Iron Treatment on MDM Cytokine Response to NTHi

We showed that the protein and gene expression of TLR4 and CD14 were downregulated in response to hemin treatment. TLR4 and CD14 act as the receptor and co-receptor, respectively, for bacterial lipopolysaccharide. TLR4 is required for the production of pro-inflammatory cytokines in response to NTHi [25]. To determine whether iron treatment reduces MDM cytokine responses to NTHi, MDMs were treated with hemin or FAC for 24 h and then exposed to live NTHi for a further 24 h. Exposure to NTHi for 24 h significantly increased the MDM production of TNF-α, IL-6 and CXCL8 above unexposed controls (# = *p* < 0.05 for all comparisons, Figure 6C–E). The 24 h treatment with hemin decreased the production of all cytokines in response to NTHi, with significance reached for TNF-α (78% reduction, *p* = 0.03), IL-6 (68% reduction, *p* = 0.008) and CXCL8 (70% reduction, *p* = 0.04) (Figure 6C–E). FAC treatment did not significantly alter cytokine production in response to NTHi exposure (*p* > 0.05, Figure S16C–E).

## 4. Discussion

We demonstrated that the number of RBCs present in the alveolar space increased in COPD patients and was significantly associated with lung macrophage iron levels. RBC numbers and lung macrophage iron levels were significantly associated with the degree of airflow obstruction. These associations suggest a role for RBC-derived iron (hemin) with regard to lung macrophage iron overload and COPD severity. To investigate this further, we evaluated the effects of hemin and FAC on macrophage phenotype and function. Hemin caused changes in macrophage phenotype markers, both at the gene expression and protein level. Furthermore, hemin impaired macrophage phagocytosis of NTHi and the production of pro-inflammatory cytokines in response to NTHi exposure. In contrast, FAC had relatively few effects on macrophage function. Overall, these results support a role for RBC-derived iron in the pathophysiology of macrophage phenotype change and dysfunction in COPD.

It has previously been reported that iron levels are increased in COPD lung macrophages [5]. Our results indicate that this is due, at least in part, to increased RBC numbers in the alveolar space of COPD patients. Other studies have focused on the detrimental effect of the dysregulation of iron homeostasis on COPD, with a focus on cigarette smoke as a likely source of increased pulmonary iron levels [4,5,14,15,16]. Our results do not support a major role for “free iron” from cigarette smoke, as the effects of the equimolar concentrations of FAC on macrophage phenotype markers and function were far less than hemin. This may be due to the unique role of hemin in TLR4 binding and stimulation of innate immunity pathways [26].

It is known that COPD macrophages have reduced phagocytosis ability. MARCO is required for the phagocytosis of bacteria [24]. We showed that MARCO and phagocytosis were downregulated in hemin-treated macrophages, implicating RBC-derived iron in the susceptibility of COPD patients to bacterial infection [27]. The dysfunction of COPD lung macrophages [28] is thought to be, in part, driven by the unique microenvironment of oxidative stress, including the potential effects of “free” iron and hemin [6], although here, we demonstrated a predominant influence of hemin over FAC. Macrophage iron levels have been associated with COPD exacerbation rates [29], providing further support for the concept that excess iron can suppress host defence against pathogens.

It has previously been reported that COPD lung macrophages have reduced cell surface expression of CD14 and CD163 [30]. We observed that CD14 and CD163 expression were reduced by hemin treatment, suggesting a role for hemin in altering COPD macrophage phenotype. Our results suggest that the surface levels of CD163 may also be reduced from the cell surface through cleavage as we saw increased levels of sCD163 in macrophage supernatants in response to hemin treatment. The reduction in surface CD163 levels may be reduced to suppress the uptake of iron to reduce cellular oxidative stress; CD163 mediates the uptake of extracellular haemoglobin when bound to haptoglobin [31,32]. HLA-DR, CD14, CD38 and CD36 are expressed at lower levels in large compared to small alveolar macrophages [28]. We observed that these four markers were downregulated following hemin treatment, suggesting that hemin treatment promotes the formation of the large macrophage subpopulation found in COPD patients [28]. In support of this, chronic iron overload has been shown to increase macrophage size and granularity [33]. Furthermore, large alveolar macrophages have reduced phagocytosis ability compared to small alveolar macrophages [28]. This subpopulation has been likened to the previously described M2c macrophage [34]. While it is accepted that previous M1/M2 categorisation is an oversimplification of macrophage phenotype, the description of this M2c population as de-activated is reflective of our findings after hemin exposure [34].

It has been reported that macrophages from COPD patients with more frequent exacerbations secrete less TNF-α and CXLC8 when exposed to NTHi or the TLR4 agonist LPS compared to macrophages from COPD patients not prone to exacerbations [35]. While we showed that hemin caused an acute burst of pro-inflammatory cytokine production, we also found that pro-inflammatory cytokine production was reduced when hemin-treated macrophages were subsequently exposed to NTHi. TLR4 and its co-receptor CD14 were downregulated following hemin treatment. These results implicate the downregulation of TLR4 expression with regard to the ability of hemin-treated macrophages to recognise NTHi and induce pro-inflammatory cytokine production. The reduced production of pro-inflammatory cytokines in response to NTHi after hemin exposure has similarities to the behaviour of COPD frequent exacerbator macrophages [35].

The role of RBCs as a potential source of iron loading in macrophages has been investigated in idiopathic pulmonary fibrosis (IPF) [36,37,38,39]. IPF lung macrophages have increased levels of iron [36,37,38,39]. These findings may be due to the vascular leakage of RBCs as a source of iron loading, with vascular resistance and pulmonary hypertension being associated with lung macrophage iron loading [37,38]. COPD patients also have increased vascular resistance and pulmonary hypertension, suggesting that similar vascular leakage mechanisms likely exist in COPD [40]. We did not investigate the link between cardiovascular abnormalities in COPD and lung macrophage iron; future investigations into this potential relationship may provide insights into the mechanisms of increased levels of lung macrophage iron.

The COVID-19 pandemic prevented the further acquisition of lung surgery samples, so we used MDMs for some experiments. While MDMs are not truly identical to lung macrophages, MDMs from COPD patients show dysfunctional behaviour compared to controls [9,41]. Using MDMs for some experiments meant that it was not possible to carry out all experiments with the same patient samples due to insufficient cell numbers. For the MDM phagocytosis and protein expression experiments, the use of MDMs allowed us to investigate the effect of iron loading on macrophages with less previous iron loading compared to lung resident cells.

When lung macrophages were isolated from the airways of lung tissue, we could not rule out the possibility that some macrophages from the population we isolated were associated with the alveolar epithelium. It is important to distinguish these macrophages from “alveolar macrophages”; hence, we used the terminology “lung macrophages” to reflect this distinction. Furthermore, given that samples were acquired from patients with suspected cancer, we cannot rule out that cancer may affect our results. To reduce any cancer-related effects, we obtained tissue from the most distal area from the tumour.

In summary, we showed that increased RBC presence in the alveolar space correlates with increased lung macrophage iron loading. We also showed red blood cell-derived iron has distinct consequences on macrophage phenotype and function. These observations suggest a potential role for red blood cell-derived iron in macrophage dysfunction and bacterial infection in COPD.

## Figures and Tables

**Table 1:** Patient demographics for all patients in the study. Kruskal–Wallis multiple comparisons test was used to test differences between groups.

	Non-Smoker	Smoker	COPD	*p* Value
*n*	26	42	74	N/A
Age (Years)	62 (19)	67 (10)	69 (7)	>0.05
Gender: Male (%)	31	29	53	<0.05
FEV1 (L)	2.7 (0.9)	2.5 (1.0)	1.7 (0.6)	<0.001
FEV1% predicted	108 (21)	99 (18)	66 (19)	<0.001
FVC (L)	3.3 (1.2)	3.0 (0.8)	3.1 (0.8)	>0.05
FEV1/FVC ratio (%)	81 (7)	80 (10)	54 (12)	<0.001
Current smokers (%)	N/A	50	47	>0.05
Pack years	N/A	33 (18)	50 (39)	<0.01
ICS usage (%)	N/A	N/A	25	N/A

Chronic obstructive pulmonary disease (COPD); forced expiratory volume in 1 s (FEV1); litres (L); forced vital capacity (FVC); inhaled corticosteroid (ICS); Not applicable (N/A) where value is 0.

## Data Availability

The data presented in this study are available from the corresponding author upon reasonable request.

## Supplementary Materials

The following are available online at https://www.mdpi.com/article/10.3390/biomedicines9121939/s1, Figure S1: Red blood cell coverage of the alveolar space image analysis example. Figure S2: Golde score scale. Figure S3: Representative images of staining of a smoker and COPD patient. Figure S4: Gating strategy for phagocytosis assay. Figure S5: Gating strategy for TLR4 and CD14 levels. Figure S6: Cytotoxicity of FAC and hemin treatment. Figure S7: Apoptosis gene expression in hemin and FAC-treated lung macrophages. Figure S8: Iron metabolism gene expression in iron-treated lung macrophages. Figure S9: Lung macrophage inflammatory marker gene expression in hemin-treated lung macrophages. Figure S10: Lung macrophage inflammatory marker gene expression in FAC-treated lung macrophages. Figure S11: Lung macrophage polarisation marker gene expression in hemin-treated lung macrophages. Figure S12: Lung macrophage polarisation marker gene expression in FAC-treated lung macrophages. Figure S13: Protein levels in FAC-treated macrophages. Figure S14: MFI of protein levels in hemin- and FAC-treated MDMs. Figure S15: Phagocytosis following NTHi stimulation in hemin- and FAC-treated non-smokers. Figure S16: Phagocytosis and cytokine response following NTHi stimulation in FAC-treated MDMs. Table S1: Subject demographics for histology patients (Figure 1). Table S2: Subject demographics for baseline lung macrophage gene expression patients (Figure 2). Table S3: Subject demographics for iron-treated gene expression patients (Figures 3, 4, and S8–S12). Table S4: Subject demographics for iron-treated apoptosis gene expression patients (Figure S7). Table S5: Subject demographics for sCD163 levels (Figures 5 and S13). Table S6: Subject demographics for phagocytosis assay patients (Figures 6, S15 and S16). Table S7: Subject demographics for COPD patient samples used for CD14 and TLR4 protein expression (Figures 5, S13 and S14). Table S8: Subject demographics for COPD patient samples used for cytokine measurement of iron pre-treated NTHi-exposed MDMs (Figures 6 and S16).
