# Supplementary material for: Red Blood Cell-Derived Iron Alters Macrophage Function in COPD
Source: Biomedicines. 2021 Dec 17;9(12):1939. doi: 10.3390/biomedicines9121939 (PMC8698324; doi:10.3390/biomedicines9121939)
Supplement: Supplementary file 1 [file biomedicines-09-01939-s001.zip › biomedicines-1482186-SI.pdf]

Online data supplement

Red Blood Cell-Derived Iron Alters Macrophage Function in COPD

James M. Baker [1,*], Molly Hammond [2], Josiah Dungwa [2], Rajesh Shah [3], Angeles Montero-Fernandez [4], Andrew Higham [1], Simon Lea [1] and Dave Singh [1,2]

[1] Division of Immunology, Immunity to Infection and Respiratory Medicine, School of Biological Sciences, Faculty of Biology, Medicine and Health, Manchester Academic Health Science Centre, The University of Manchester and Manchester University NHS Foundation Trust, Manchester M13 9PL, UK; andrew.higham@manchester.ac.uk (A.H.); simon.lea@manchester.ac.uk (S.L.); dsingh@meu.org.uk (D.S.)

[2] Medicines Evaluation Unit, Manchester University NHS Foundation Trust, Manchester M23 9QZ, UK; mollyhammond7@icloud.com (M.H.); jdungwa@meu.org.uk (J.D.)

[3] Department of Thoracic Surgery, Manchester University Hospital NHS Foundation Trust, Manchester M13 9WL, UK; rajesh.shah@mft.nhs.uk

[4] Department of Histopathology, Manchester University Hospital NHS Foundation Trust, Manchester M13 9WL, UK; angeles.montero@mft.nhs.uk

* Correspondence: james.baker-6@postgrad.manchester.ac.uk; Tel.: +44-16-1219-5920

METHODS

Study subjects

COPD was diagnosed based on a smoking history of ≥10 pack years and GOLD criteria (1). Controls were smokers (S) without airflow limitation or non-smokers (NS). Ex-smokers were defined as individuals who had stopped smoking for ≥1 year.

Lung macrophage isolation

Lung tissue was obtained through surgery from patients with suspected cancer. Tissue was then taken from areas most distal from the tumour. Resected lung tissue airways were perfused with 0.1 M NaCl. The subsequent fluid was then centrifuged for 10 min at 400× *g* at room temperature. The cell pellet was then resuspended in RPMI-1640 media (Sigma-Aldrich, Poole, UK). The cell suspension was then layered over Ficoll-Paque (GE Healthcare, Buckinghamshire, UK) and was centrifuged at 400× *g* for 30 min at room temperature with the no-brake setting. The interphase was removed from the Ficoll suspension using a pasture pipette and was washed with RMPI-1640 with 1% Penicillin Streptomycin Solution, 1% L-Glutamine and 10% Fetal Calf Serum. Cell counting was performed using a haemocytometer (Neubauer haemocytometer) and Trypan Blue exclusion dye.

Tissue preparation for IHC

Tissue blocks were obtained from patients undergoing surgical resection for lung cancer as previously described (2). Patients were diagnosed with COPD on the basis of spirometry values and symptoms (3).

Tissue blocks were formalin fixed and paraffin embedded where they were then cut into 4-µM sections and were mounted on a glass slide. As with all tissue sections described herein, the sections were deparaffinised in xylene and rehydrated in graded alcohols. Each tissue block was validated, with sections undergoing Haematoxylin and Eosin staining to assess the fixing and embedding process.

Quantification of iron and ferritin in macrophages

Tissue was stained with Perls Prussian Blue. Intensity was quantified using a previously described semi-quantitative scale, Golde Score (3). Golde score was calculated through assigning a staining intensity score (0–4) for each macrophage using the scale shown in Fig. S2. The lowest number of macrophages analysed in a patient was 68. Values were then normalised to 100 macrophages per patient to give Golde Score. The same tissue was then stained by dual immunofluorescent labelling of anti-human FTH-1 and anti-human CD68 (representative images can be seen in Figure S3 of a smoker (A, B, E, F) and COPD patient (C, D, G, H). CD68 staining was used to determine quantity and distribution of lung macrophages. Measurements for iron and ferritin levels in each were then collected for lung macrophage. Ferritin levels were assessed to investigate the mechanism of iron storage in lung macrophages in COPD. Due to ubiquitous expression of ferritin in lung macrophages, ferritin levels were assessed based on intensity of staining to define lung macrophages as ferritin^{high} or ferritin^{low}.

Perls Prussian Blue

Perls Prussian Blue staining was carried out according to the manufacturer's instructions (Sigma-Aldrich, Germany). Equal parts Ferrocyanide and Hydrochloric acid was added to the tissue for 10 min followed by washing in distilled water. Pararosaniline was then added for 5 min followed by washing in distilled water. Coverslips were then adding using aqueous mount. A slide with a numbered grid (England Finder, Agar Scientific, Essex, UK) was placed under the tissue slide to enable location of the same field of view for subsequent CD68 and FTH-1 immunofluorescent staining (described below). Slides were then imaged using a Nikon Eclipse 80i microscope (Nikon UK Ltd., Surrey, UK) equipped with a QImaging digital camera (Teledyne Photmetrics, Tucson, AZ, USA).

CD68 and Ferritin Immunofluorescence

Antigen retrieval was carried out in a microwave in pH8 buffer for 15 min. The slides were then allowed to cool for 20 min and subsequently washed in TBST for 5 min. Blocking was then undertaken in 1.5% horse serum for 30 min. Mouse anti-human CD68 primary antibody (1:200; cat# Agilent, M087629) and rabbit anti-human ferritin (1:600; cat# abx100093, Abbexa) diluted in antibody diluent and incubated on the slide for 1 hour and 30 min. Slides were washed twice for 5 minute washes in TBST. Donkey anti-mouse Alexa Fluor 488 (1:200; cat# A21202, Thermo Fisher Scientific) and goat anti-rabbit Alexa Fluor 568 (1:200; cat# A10037, Thermo Fisher Scientific) in TBS. Slides were then washed twice in TBST for 5 min. Vector® TrueVIEW™ Autofluorescence Quenching Kit (VectorLabs, Peterborough, UK) was

then used for 4 min and was subsequently mounted with one drop of VECTASHIELD® Antifade Mounting Medium with DAPI (VectorLabs, Peterborough, UK) and cover-slipped. The same fields of view used for Perls staining were located using the slide with a numbered grid (England Finder, Agar Scientific, Essex UK). Images were obtained using a Nikon Eclipse 80i microscope (Nikon UK Ltd, Surrey, UK) equipped with a QImaging digital camera (Teledyne Photmetrics, Tucson, AZ, USA). All analysis was blinded and underwent validation by an independent observer with less than 10% variance.

MTT assay

Cytotoxicity was assessed with an MTT assay (Abcam) ab211091. Assay was carried out according to manufacturer's instructions. Lung macrophages were seeded overnight and were subsequently treated with FAC, hemin or were untreated controls for 2, 4, 6 and 24 h. RPMI medium (10% FBC, 2 mM glutamine, penicillin and streptomycin)/iron was removed and cells were washed with DPBS. 50 µL of RPMI without FBS was added as well as 50 µL of MTT working solution. The cells were then incubated for 3 h at 37 ^{0}C and 5% CO_2. 150 µL of MTT solvent was added and was mixed on a shaker for 15 min. The plate was then read at 590 nm on using a POLAstar Omega (BMG LABTECH) plate reader.

Gene expression

Transferrin receptor 1 (TFRC), Ferroportin (FPN), Iron-responsive element-binding protein (IRP1), Iron-responsive element-binding protein 2 (IRP2), Ferritin heavy chain 1 (FTH-1), HO-1, inflammatory genes; TNF-α, CXCL8, IL10, macrophage polarisation markers; CD36, CD206,

CD163, HLA-DR, CD38, Toll-like receptor 4 (TLR4), CD14, MARCO or apoptosis associated genes: B-cell lymphoma 2 (BCL2), Induced myeloid leukemia cell differentiation protein Mcl-1 (MCL1), BCL2-like 1 (BCL2L1), BCL2 homologous antagonist/killer (BAK1), BCL2 associated x protein (BAX) and the endogenous control Glyceraldehyde 3-phosphate dehydrogenase (GAPDH).

sCD163 release in lung macrophages

Lung macrophages were treated with hemin or FAC (50, 100 or 200 µM) for 24 h. Supernatant sCD163 levels were measured according to manufacturers' instructions (R&D systems, UK eBioscience, San Diego).

Culture, opsonisation and phagocytosis of bacteria

Non-typeable *Haemophilus influenzae* (NTHi) (NCTC 12699) was cultured in conditions previously described (4). NTHi CFU/mL was calculated using Miles Misra. The remaining NTHi stock was heat killed at 90 ^{o}C for 30 min before being stored at −80 ^{o}C. The calculated CFU/mL from the Miles Misra was then used to calculate the volume of bacterial suspension required for an MOI of 10:1. Peripheral blood was collected in an SST vacutainer (BD Biosciences) from 5 healthy never smokers and processed according to manufacturer's instructions. The serum was collected and heat-inactivated at 56 ^{o}C for 30 min, pooled and stored at -80 ^{o}C. An aliquot of NTHi was thawed for each experiment and was labelled with Alexa Fluor™ 488 NHS Ester (Thermofisher, CAT: A20000) for 30 min before washing twice with 1% heat inactivated fetal bovine serum and 0.1% Sodium Azide in PBS (wash buffer). NTHi was then opsonised using

10% heat-inactivated human serum for 30 min at room temperature on a shaker. Opsonisation was confirmed using this pooled serum previously (4). NTHi was then washed and then resuspended in 2% BSA. Iron-media supernatants were removed and monocyte derived macrophages wells were washed before 3 hour incubation with NTHi in RMPI.

Monocyte-derived macrophages were removed from the plate by manual dissociation. For phagocytosis experiments, monocyte-derived macrophages were incubated with trypan blue for 1 min before washing twice in wash buffer. Finally, cell pellets were resuspended in 300 µL of wash buffer and acquired on FACS Canto II using FACS DIVA software. Data was then analysed using Flowjo software (Treestar, USA). Monocyte derived macrophage phagocytosis and phenotype marker expression gating strategy is shown in Figures S4 and S5.

For cytokine production experiments, MDMs were exposed to 24 h of hemin or FAC before the supernatants were washed away. MDMs were then exposed to live NTHi for a further 24 h. NTHi MOI was estimated using Miles Misra as described above to achieve an MOI of approximately 1:50. Phagocytosis experiments were carried out with heat killed bacteria as described above, with an MOI of 1:10.

Statistics

A repeated measures 2-way ANOVA with the Greenhouse-Geisser correction and a Dunnetts multiple comparison test was carried out to test for cytotoxicity of iron treatments. Where

not indicated, post-hoc multiple comparisons tests were carried out compared to untreated controls.

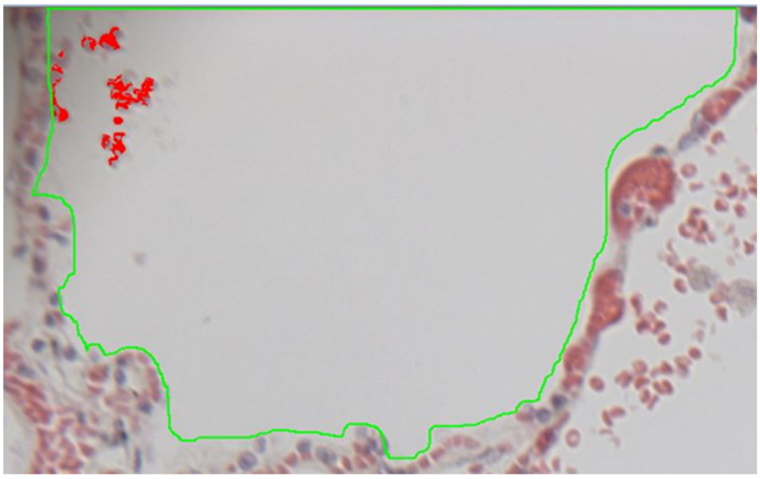

Figure S1. Red blood cell coverage of the alveolar space image analysis example. Green outline was drawn to determine alveolar area. Superimposed red colour was generated to determine red blood cell area.

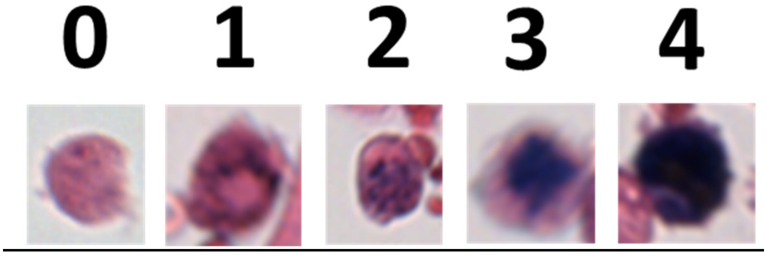

Figure S2. Golde Score scale. Semi-quantitative scale used to grade lung macrophages iron levels based on staining intensity/coverage. 0: no staining. 1: faint staining in part of the cell (<50%). 2: stronger staining in most (~50%) of the cell. 3: strong staining in nearly the whole cell (blue). 4: strong staining in the whole cell (black).

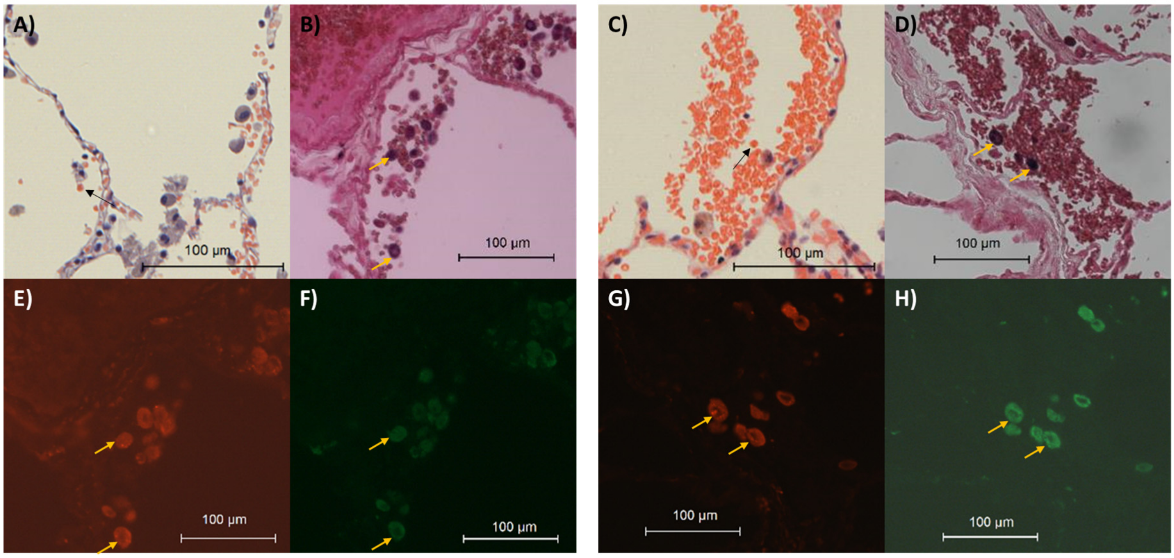

Figure S3. Representative images of staining of a smoker and COPD patient. H&E staining carried out to quantify red blood cell (RBC) coverage in a (**A**) smoker and a (**C**) COPD patient. Perls staining carried out to measure iron levels in a (**B**) smoker and (**D**) COPD patient. CD68 staining in a (**F**) smoker and a (**H**) COPD patient to locate lung macrophages. FTH-1 staining in (**E**) a smoker and (**G**) a COPD patient to quantify ferritin levels in lung macrophages. Yellow arrows indicate the same lung macrophages across different stains. Black arrows indicate RBCs.

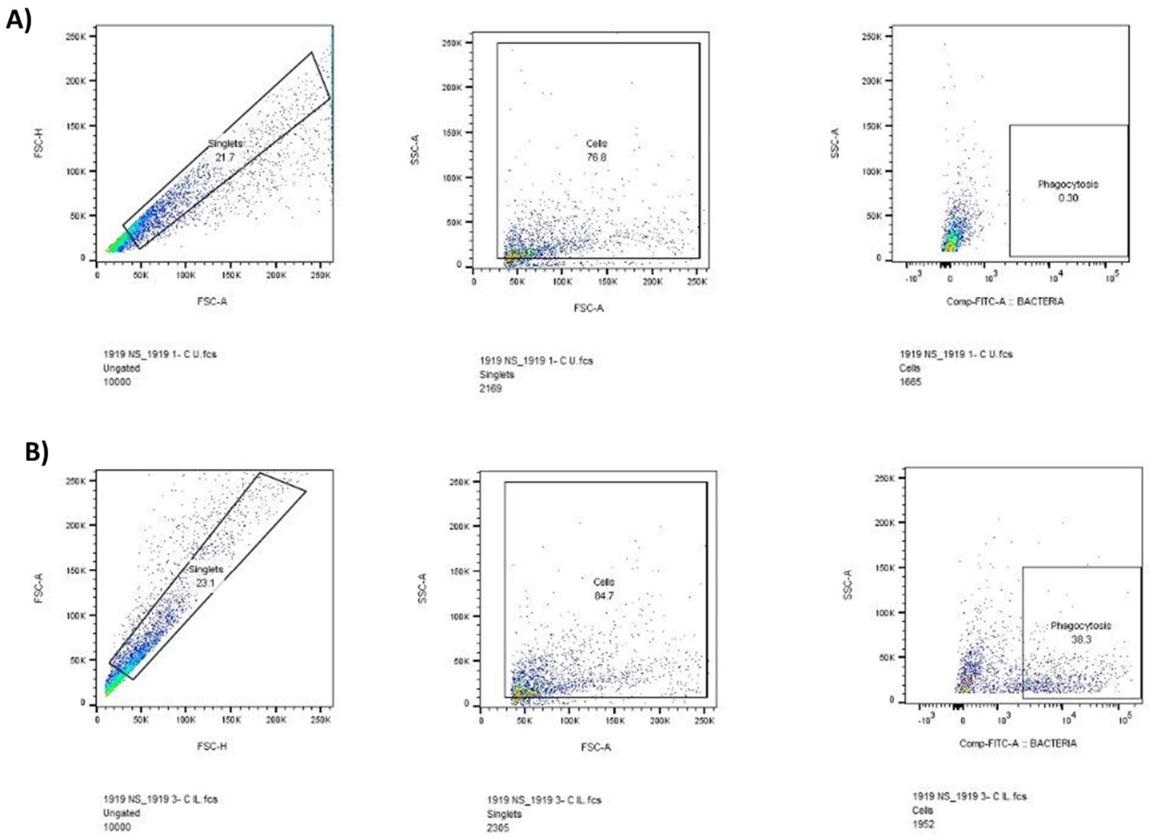

Figure S4. Gating strategy for phagocytosis assay. Monocyte derived macrophages (MDMs) were pre-treated with hemin or ferric ammonium citrate (FAC) for 24 h followed by challenge with Non-typeable *Haemophilus influenzae* (NTHi). Gating strategy illustrated for phagocytosis assay in (**A**) unstimulated and (**B**) NTHi stimulated MDMs.

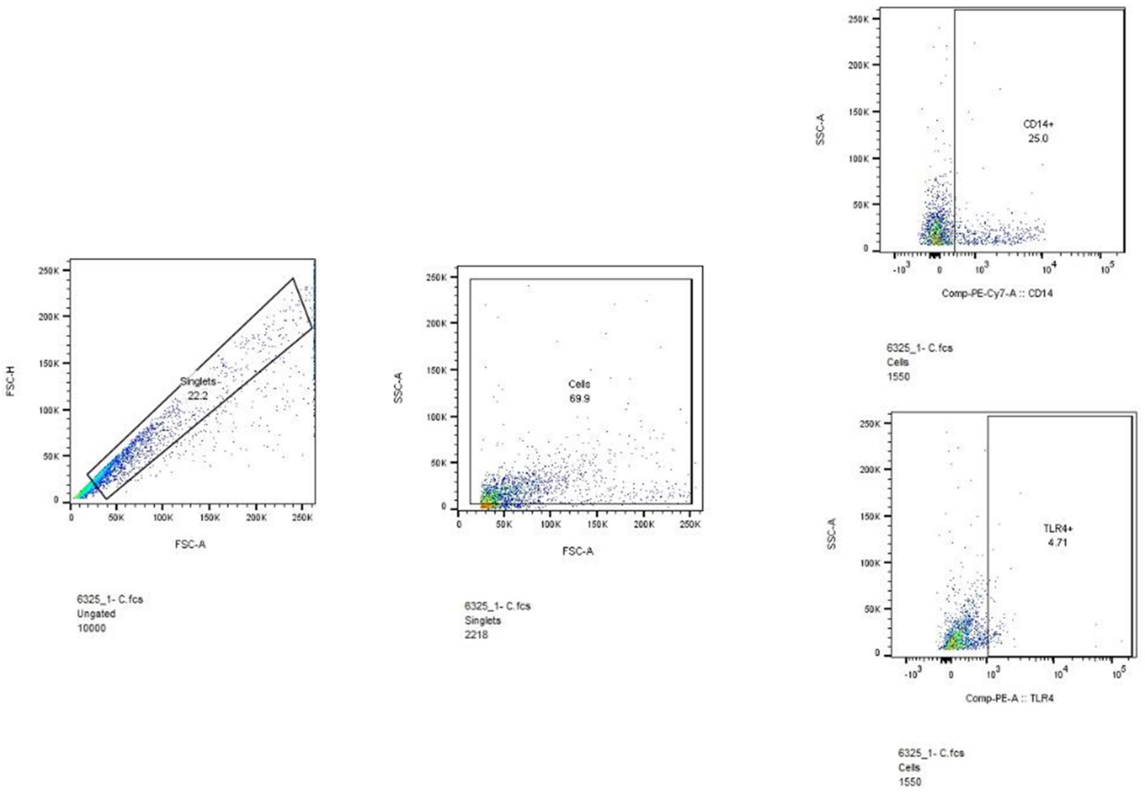

Figure S5. Gating strategy for TLR4 and CD14 levels. Monocyte derived macrophages (MDMs) were treated with hemin or ferric ammonium citrate (FAC) for 24 h.

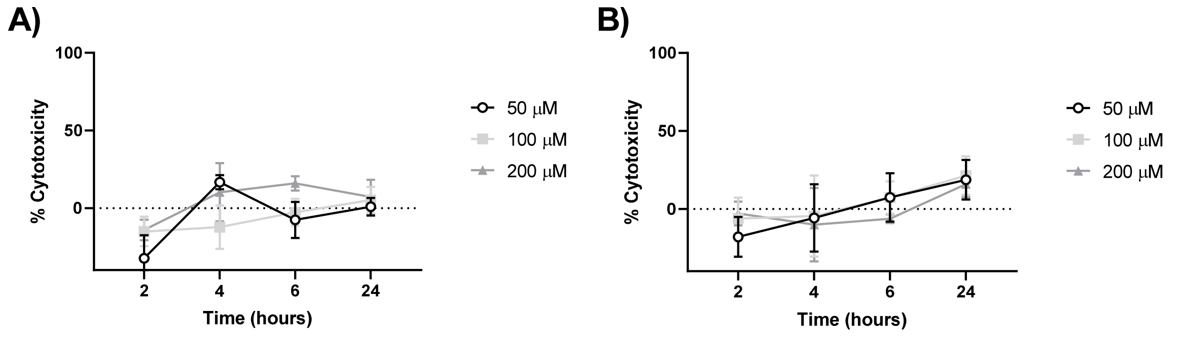

Figure S6. Cytotoxicity of FAC and hemin treatment. Lung macrophages were cultured with (**A**) Hemin (50, 100 and 200 μM) or (**B**) ferric ammonium citrate (FAC) (50, 100 and 200 μM) for 2, 4, 6 and 24 h in smokers and COPD patients (n = 6). Percentage of cytotoxicity was determined through an MTT assay.

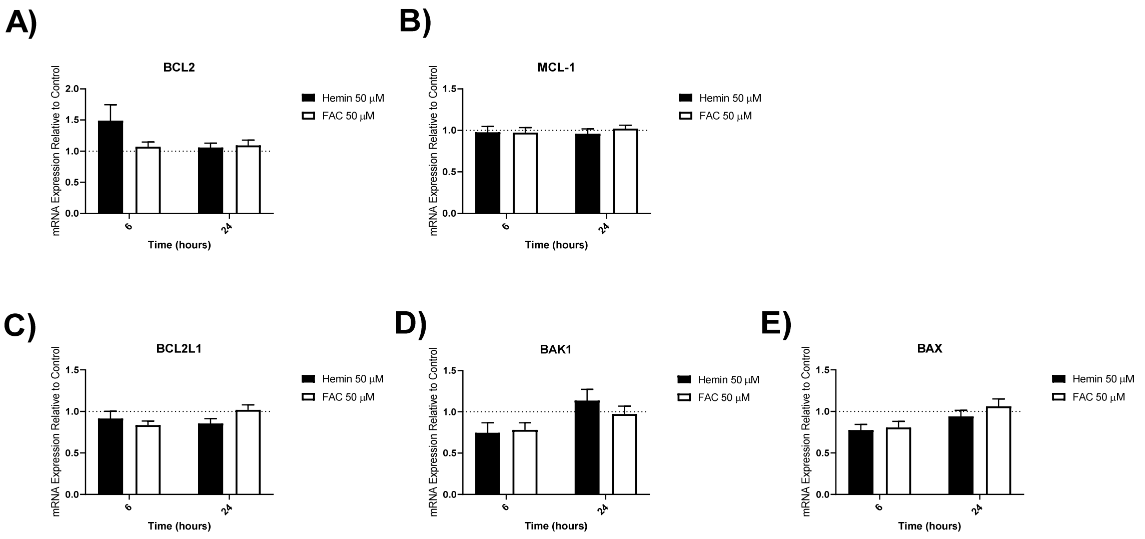

Figure S7. Apoptosis gene expression in hemin and FAC treated lung macrophages. Lung macrophages were cultured for 6 and 24 h treated with 50 μM of hemin or FAC. (**A**) BCL2, (**B**) MCL-1, (**C**) BCL2L1, (**D**) BAK1 and (**E**) BAX expression was measured. Real-time qPCR was used to assess levels of gene expression after 6 and 24 h in smokers and COPD patients (n = 10). Gene expression shown was plotted as means relative to GAPDH and untreated control for each patient ($2^{-\Delta\Delta Ct}$) ±SEM. Dotted line at 1 indicates untreated control.

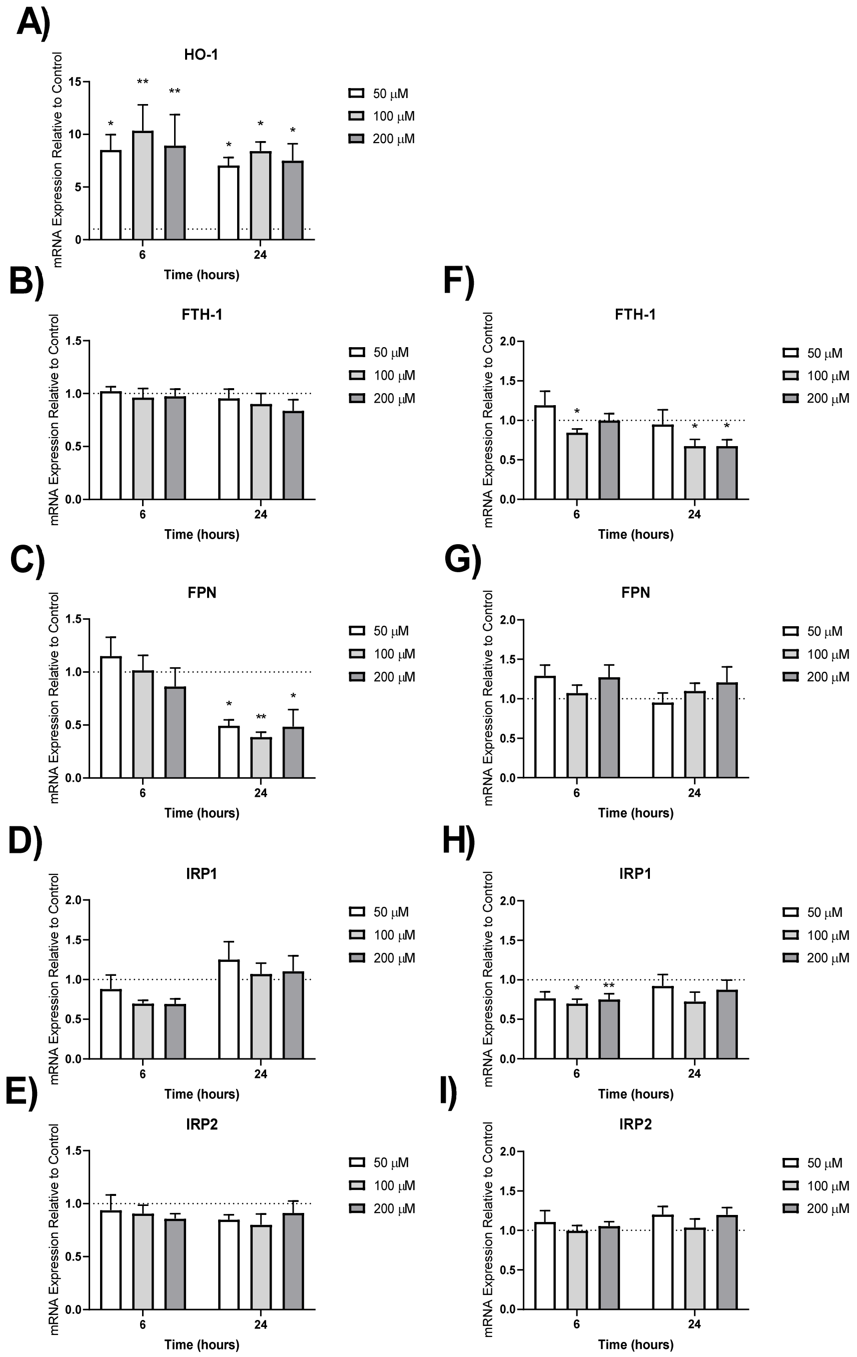

Figure S8. Iron metabolism gene expression in iron treated lung macrophages. HO-1, FTH-1, FPN, IRP1 and IRP2 expression in lung macrophages treated with 50 μM, 100 μM or 200 μM of hemin (**A**–**E**) or ferric ammonium citrate (FAC) (**F**–**I**). Real-time qPCR was used to assess levels of gene expression after 6 and 24 h. Gene expression shown was plotted as means relative to GAPDH and untreated control for each patient (2−ΔΔCt) ±SEM in smokers and

COPD patients (n = 6). Dotted line at 1 indicates untreated control. One-way ANOVAs were carried out with a post hoc Dunnetts multiple comparisons test against time-matched untreated controls. * = $p < 0.05$, ** = $p < 0.01$.

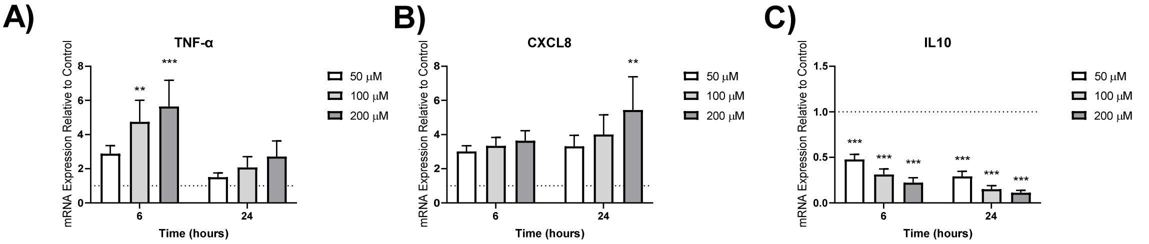

Figure S9. Lung macrophage inflammatory markers gene expression in hemin treated lung macrophages. TNF-α, CXCL8 and IL10 expression in lung macrophages treated with 50 µM, 100 µM or 200 µM of hemin (**A**–**C**). Real-time qPCR was used to assess levels of gene expression after 6 and 24 h in smokers and COPD patients (*n* = 6). Gene expression shown was plotted as means relative to GAPDH and untreated control for each patient ($2^{-\Delta\Delta Ct}$) ±SEM. Dotted line at 1 indicates untreated control. One-way ANOVAs were carried out with a post hoc Dunnetts multiple comparisons test against time-matched untreated controls. ** = $p < 0.01$, *** = $p < 0.001$.

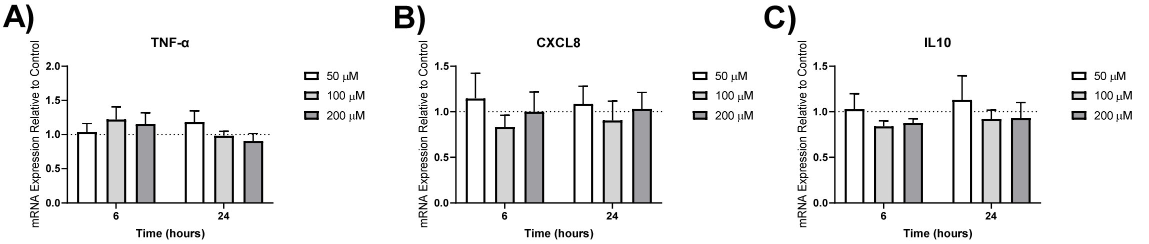

Figure S10. Lung macrophage inflammatory markers gene expression in FAC treated lung macrophages. TNF-α, CXCL8 and IL10 expression in lung macrophages treated with 50 µM,

100 µM or 200 µM of FAC (**A**–**C**). Real-time qPCR was used to assess levels of gene expression after 6 and 24 h in smokers and COPD patients (*n* = 6). Gene expression shown was plotted as means relative to GAPDH and untreated control for each patient ($2^{-\Delta\Delta Ct}$) ±SEM. Dotted line at 1 indicates untreated control. One-way ANOVAs were carried out with a post hoc Dunnetts multiple comparisons test against time-matched untreated controls.

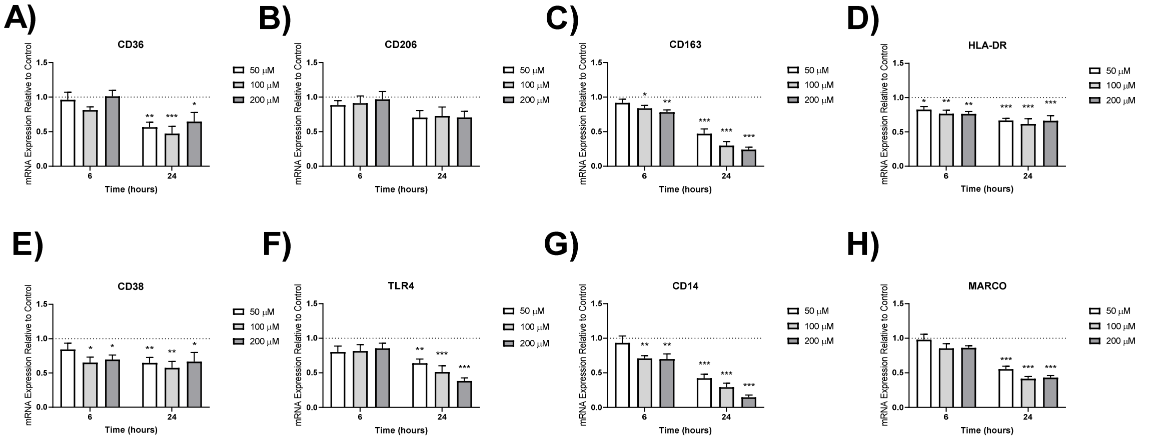

Figure S11. Lung macrophage polarisation marker gene expression in hemin treated lung macrophages. CD36, CD206, CD163, HLA-DR, CD38, TLR4, CD14, and MARCO expression in lung macrophages treated with 50 µM, 100 µM or 200 µM of hemin (**A**–**H**). Real-time qPCR was used to assess levels of gene expression after 6 and 24 h in smokers and COPD patients (n = 6). Gene expression shown was plotted as means relative to GAPDH and untreated control for each patient ($2^{-\Delta\Delta Ct}$) ±SEM. Dotted line at 1 indicates untreated control. One-way ANOVAs were carried out with a post hoc Dunnetts multiple comparisons test against time-matched untreated controls. * = $p < 0.05$, ** = $p < 0.01$, *** = $p < 0.001$.

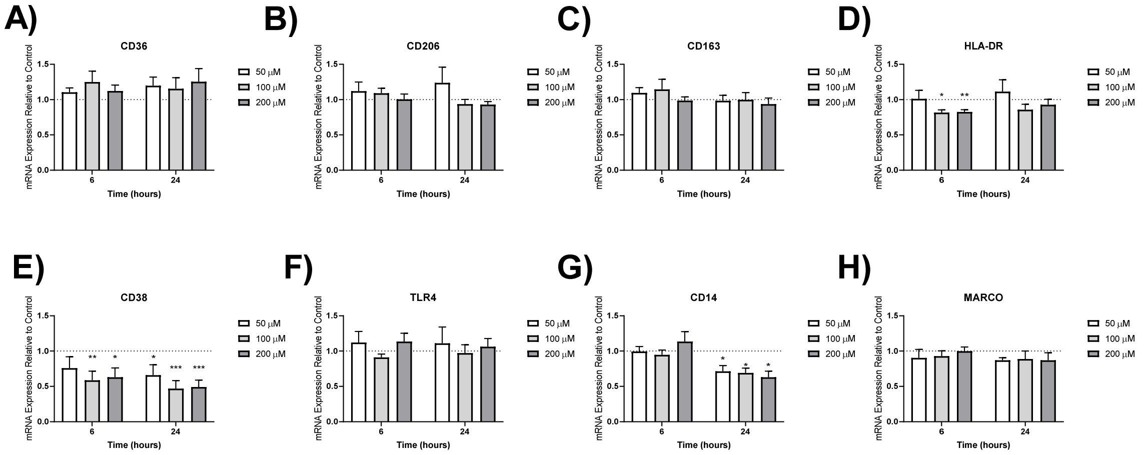

Figure S12. Lung macrophage polarisation marker gene expression in FAC treated lung macrophages. CD36, CD206, CD163, HLA-DR, CD38, TLR4, CD14, and MARCO expression in lung macrophages treated with 50 µM, 100 µM or 200 µM of ferric ammonium citrate (FAC) (**A**–**H**). Real-time qPCR was used to assess levels of gene expression after 6 and 24 h in smokers and COPD patients (n = 6). Gene expression shown was plotted as means relative to GAPDH and untreated control for each patient ($2^{-\Delta\Delta Ct}$) ±SEM. Dotted line at 1 indicates untreated control. One-way ANOVAs were carried out with a post hoc Dunnetts multiple comparisons test against time-matched untreated controls. * = $p < 0.05$, ** = $p < 0.01$, *** = $p < 0.001$.

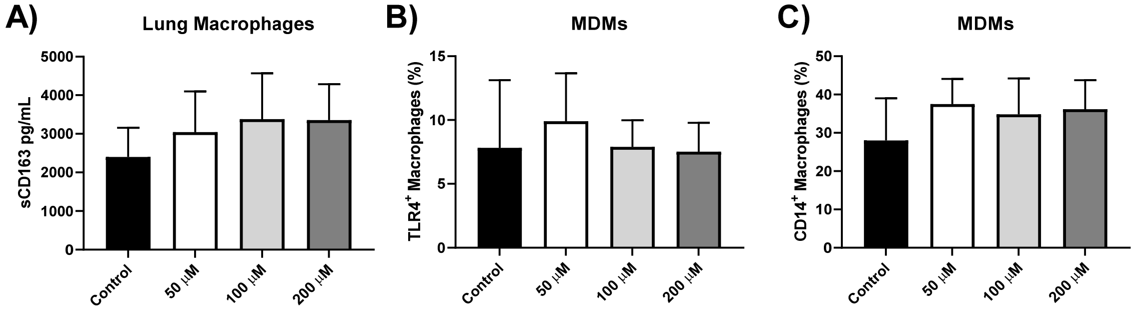

Figure S13. Protein levels in FAC treated macrophages. (**A**) sCD163 levels in supernatants from lung macrophages treated with 50 µM, 100 µM or 200 µM of ferric ammonium citrate

(FAC). ELISA was used to assess levels of sCD163 after 24 h in smokers and COPD patients (*n* = 8). Percentage of (**B**) TLR4 and (**C**) CD14 positive MDMs after 24 hour treatment with 50, 100 or 200 µM of FAC in COPD patients (*n* = 5). One-way ANOVAs were carried out with a post hoc Dunnetts multiple comparisons test against time-matched untreated controls. Data presented as ±SEM.

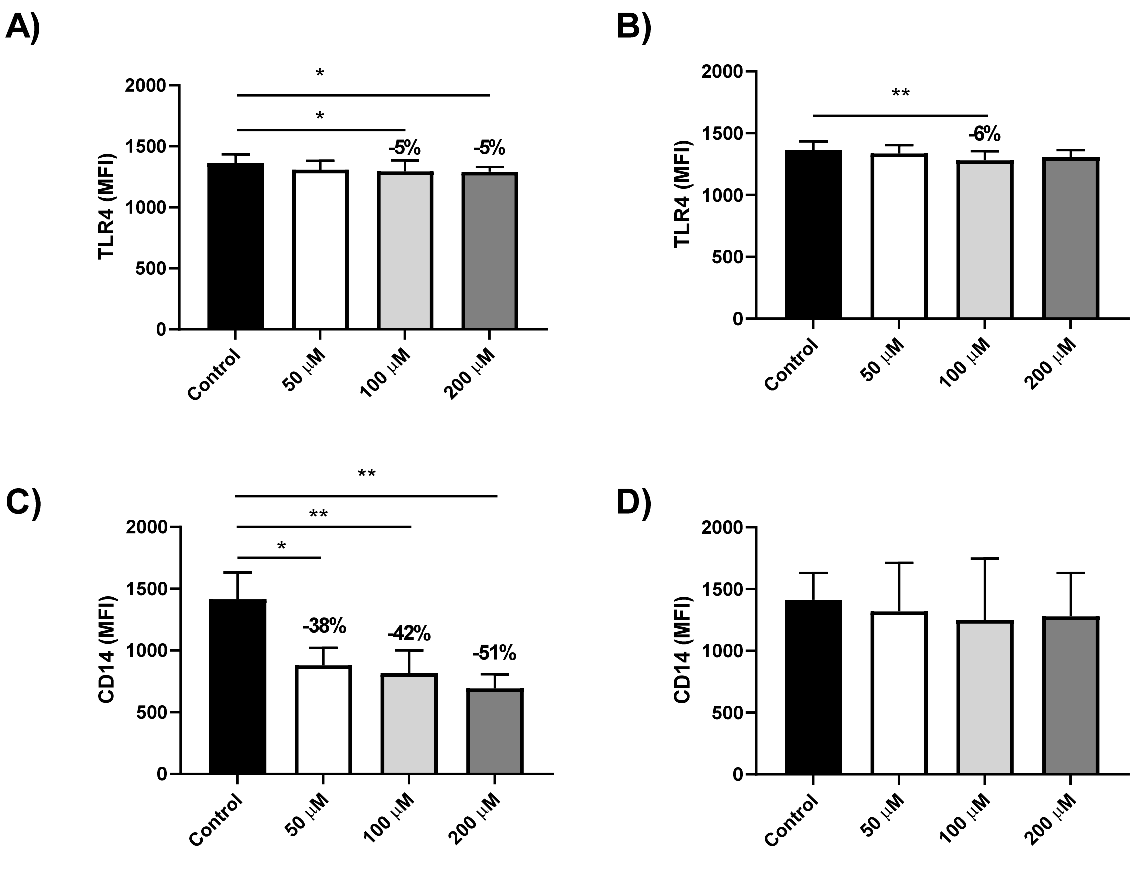

Figure S14. MFI of protein levels in hemin and FAC treated MDMs. Monocyte derived macrophages (MDMs) were treated with 50 µM, 100 µM or 200 µM of hemin or ferric ammonium citrate (FAC) in COPD patients (*n* = 5). MFI of TLR4 in (**A**) hemin and (**B**) FAC was measured (*n* = 5). CD14 MFI was also measured in (**C**) hemin and (**D**) FAC. One-way ANOVAs were carried out with a post hoc Dunnetts multiple comparisons test against time-matched

untreated controls. Data presented as ±SEM. Relative percentage changed indicated on graph. * = $p < 0.05$, ** = $p < 0.01$.

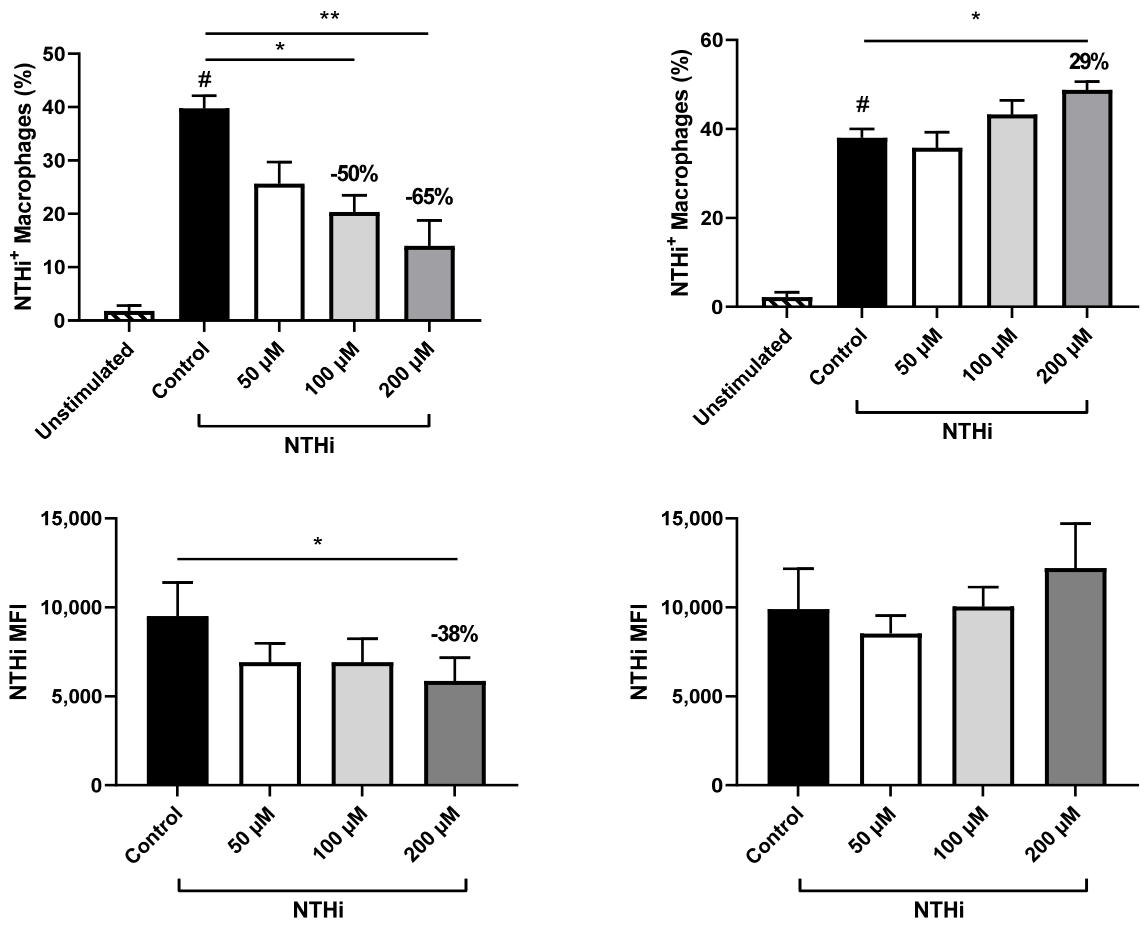

Figure S15. Phagocytosis following NTHi stimulation in hemin and FAC treated non-smokers. Non-smoker (NS) monocyte derived macrophages (MDMs) were pre-treated with 50 μM, 100 μM or 200 μM of hemin and ferric ammonium citrate (FAC) for 24 h followed by exposure to labelled Non-typeable *Haemophilus influenzae* (NTHi) for 3 h (n = 6). Phagocytosis of FITC labelled NTHi in NS MDMs pre-treated with (**A**) hemin and (**B**) FAC was measured using flow cytometry. MFI of NS MDMs pre-treated with (**C**) hemin and (**D**) FAC was measured using flow cytometry. One-way ANOVAs were carried out with a post hoc Dunnetts multiple comparisons test against untreated/stimulated controls. Relative percentage changed

indicated on graph. * = $p < 0.05$, ** = $p < 0.01$. Unstimulated vs stimulated control *t*-test # = $p < 0.05$.

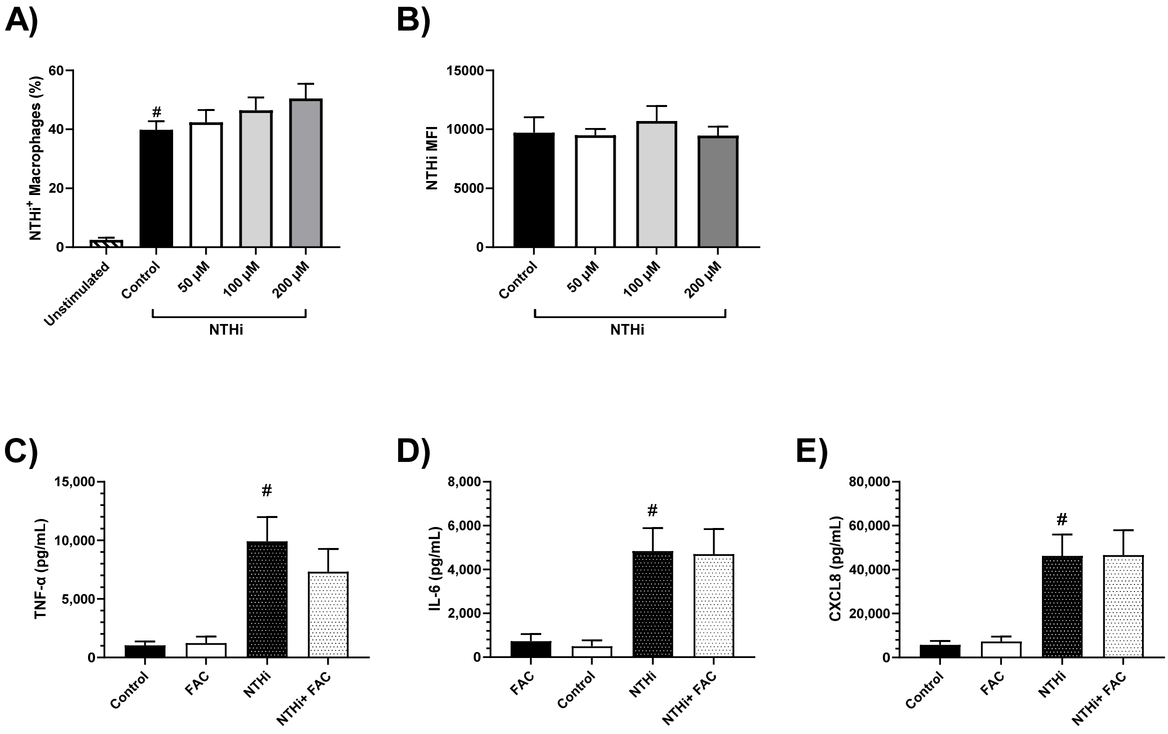

Figure S16. Phagocytosis and cytokine response following NTHi stimulation in FAC treated MDMs. Monocyte derived macrophages (MDMs) were pre-treated with 50 µM, 100 µM or 200 µM of ferric ammonium citrate (FAC) for 24 h followed by exposure to labelled Non-typeable *Haemophilus influenzae* (NTHi) for 3 h. (**A**) Phagocytosis of FITC labelled NTHi in FAC pre-treated COPD MDMs was measured using flow cytometry (n = 7). (**B**) MFI of COPD MDMs with labelled NTHi internalised. Macrophages pre-treated with 200 µM of FAC for 24 h were subsequently stimulated for a further 24 h with live bacteria (n = 9). Cytokine levels production was measured for (**C**) TNF-α, (**D**) IL-6 and (**E**) CXCL8. One-way ANOVAs were carried out with a post hoc Dunnetts multiple comparisons test against untreated/stimulated controls (**A**,**B**). Unstimulated vs stimulated control t-test # = $p < 0.05$.

Table S1. Subject demographics for histology patients (Figure 1). Forced expiratory volume in one second (FEV1), Litres (L), Forced vital capacity (FVC), Inhaled corticosteroid (ICS). Kruskal-Wallace Multiple Comparisons Test was used to test differences between groups.

	Non-Smoker	Smoker	COPD	*p* Value
**n**	11	15	32	N/A
**Age (Years)**	71(10)	63 (12)	69 (8)	>0.05
**Gender: Male (%)**	27	33	71	<0.01
**FEV_1 (L)**	2.2 (0.4)	2.6 (1.6)	1.8 (0.7)	<0.05
**FEV1 % predicted**	101 (12)	92 (16)	65 (21)	<0.001
**FVC (L)**	2.7 (0.7)	2.8 (0.7)	3.2 (0.9)	>0.05
**FEV1/FVC Ratio (%)**	79 (6)	79 (6)	54 (12)	<0.001
**Current smokers (%)**	N/A	40	47	>0.05
**Pack years**	N/A	31 (24)	50 (42)	<0.05
**ICS usage (%)**	N/A	N/A	23	N/A

Table S2. Subject demographics for baseline lung macrophage gene expression patients (Figure 2). Forced expiratory volume in one second (FEV1), Litres (L), Forced vital capacity (FVC), Inhaled corticosteroid (ICS). Kruskal-Wallace Multiple Comparisons Test was used to test differences between groups.

	Non-Smoker	Smoker	COPD	*p* Value
**n**	8	24	27	N/A
**Age (Years)**	71 (13)	71 (7)	68 (8)	>0.05
**Gender: Male (%)**	13	36	27	>0.05
**FEV_1 (L)**	2.5 (1.0)	2.2 (0.5)	16.05	<0.001
**FEV1 % predicted**	126 (24)	100 (21)	70 (13)	<0.001
**FVC (L)**	3.2 (1.1)	2.7 (0.7)	3.0 (0.8)	>0.05
**FEV1/FVC Ratio (%)**	83 (9)	80 (14)	55 (9)	<0.001
**Current smokers (%)**	N/A	40	43	>0.05
**Pack years**	N/A	36 (14)	54 (45)	>0.05
**ICS usage (%)**	N/A	N/A	8	N/A

Table S3. Subject demographics for iron treated gene expression patients (Figure 3 and 4, Figure S8, S9, S10, S11 and S12). Forced expiratory volume in one second (FEV1), Litres (L), Forced vital capacity (FVC), Inhaled corticosteroid (ICS).

Demography	Ex-Smoker/COPD
**n**	1/5
**Age (Years)**	73 (5)
**Gender: Male (%)**	50
**FEV_1 (L)**	1.9 (1.0)
**FEV1 % predicted**	79 (20)
**FVC (L)**	3.1 (1.2)
**FEV1/FVC Ratio (%)**	65 (14)
**Current smokers (%)**	66
**Pack years**	59
**ICS usage (%)**	17

Table S4. Subject demographics for iron treated apoptosis gene expression patients (Figure S7). Forced expiratory volume in one second (FEV1), Litres (L), Forced vital capacity (FVC), Inhaled corticosteroid (ICS). An unpaired t-test Comparisons Test was used to test differences between groups.

Demography	Smoker	COPD	*p* Value
**n**	5	5	N/A
**Age (Years)**	66 (9)	68 (6)	>0.05
**Gender: Male (%)**	20	40	>0.05
**FEV_1 (L)**	2.6 (0.8)	1.6 (0.4)	<0.05
**FEV1 % predicted**	102 (20)	66 (20)	<0.05
**FVC (L)**	3.4 (1.1)	3.0 (0.3)	>0.05
**FEV1/FVC Ratio (%)**	75 (6.0)	57 (16)	<0.05
**Current smokers (%)**	40	60	>0.05
**Pack years**	37 (13)	42 (10)	>0.05
**ICS usage (%)**	N/A	N/A	N/A

Table S5. Subject demographics for sCD163 levels (Figure 5 and Figure S13). Forced expiratory volume in one second (FEV1), Litres (L), Forced vital capacity (FVC), Inhaled corticosteroid (ICS).

Demography	Smoker/COPD
**n**	3/5
**Age (Years)**	68 (7)
**Gender: Male (%)**	38
**FEV_1 (L)**	2.2 (0.8)
**FEV1 % predicted**	90 (24)
**FVC (L)**	3.4 (0.8)
**FEV1/FVC Ratio (%)**	72 (10)
**Current smokers (%)**	63
**Pack years**	46 (15)
**ICS usage (%)**	13

Table S6. Subject demographics for phagocytosis assay patients (Figure 6 and Figure S15 and S16). Forced expiratory volume in one second (FEV1), Litres (L), Forced vital capacity (FVC), Inhaled corticosteroid (ICS). An unpaired t-test Comparisons Test was used to test differences between groups.

Demography	NS	COPD	*p* Value
**n**	6	7	N/A
**Age (Years)**	36 (12)	69 (5)	<0.001
**Gender: Male (%)**	66	43	>0.05
**FEV_1 (L)**	3.8 (0.5)	1.6 (0.9)	<0.001
**FEV1 % predicted**	95 (9)	61 (28)	<0.05
**FVC (L)**	5.0 (1.0)	3.5 (1.0)	>0.05
**FEV1/FVC Ratio (%)**	78 (5)	48 (17)	<0.01
**Current smokers (%)**	N/A	29	N/A
**Pack years**	N/A	34 (10)	N/A
**ICS usage (%)**	N/A	43	N/A

Table S7. Subject demographics for COPD patients MDM samples used for CD14 and TLR4 protein expression (Figure 5 and Figure S13 and S14). Forced expiratory volume in one second (FEV1), Litres (L), Forced vital capacity (FVC), Inhaled corticosteroid (ICS).

Demography	COPD
**n**	5
**Age (Years)**	68 (3)
**Gender: Male (%)**	40
**FEV_1 (L)**	1.9 (1.0)
**FEV1 % predicted**	69 (29)
**FVC (L)**	3.6 (1.4)
**FEV1/FVC Ratio (%)**	56 (13)
**Current smokers (%)**	60
**Pack years**	43 (11)
**ICS usage (%)**	40

Table S8. Subject demographics for non-smoker COPD patients samples used for cytokine measurement of iron pre-treated NTHi exposed MDMs (Figure 6 and Figure S16). Forced expiratory volume in one second (FEV1), Litres (L), Forced vital capacity (FVC), Inhaled corticosteroid (ICS).

Demography	NS/COPD
**n**	2/5
**Age (Years)**	60 (17)
**Gender: Male (%)**	63
**FEV_1 (L)**	2.5 (1.1)
**FEV1 % predicted**	80 (22)
**FVC (L)**	3.9 (1.2)
**FEV1/FVC Ratio (%)**	62 (13)
**Current smokers (%)**	25
**Pack years**	32 (22)
**ICS usage (%)**	38

References

1. Vogelmeier CF, Criner GJ, Martinez FJ, Anzueto A, Barnes PJ, Bourbeau J, et al. Global Strategy for the Diagnosis, Management, and Prevention of Chronic Obstructive Lung Disease 2017 Report. GOLD Executive Summary. American journal of respiratory and critical care medicine. 2017;195(5):557-82.
2. Lea S, Plumb J, Metcalfe H, Spicer D, Woodman P, Fox JC, et al. The effect of peroxisome proliferator-activated receptor-gamma ligands on in vitro and in vivo models of COPD. The European respiratory journal. 2014;43(2):409-20.
3. Plumb J, Smyth LJC, Adams HR, Vestbo J, Bentley A, Singh SD. Increased T-regulatory cells within lymphocyte follicles in moderate COPD. The European respiratory journal. 2009;34(1):89-94.
4. Higham A, Scott T, Li J, Gaskell R, Dikwa AB, Shah R, et al. Effects of corticosteroids on COPD lung macrophage phenotype and function. Clinical science (London, England : 1979). 2020;134(7):751-63.
